# Genetic Simulation Resources: a website for the registration and discovery of genetic data simulators

**DOI:** 10.1093/bioinformatics/btt094

**Published:** 2013-02-23

**Authors:** Bo Peng, Huann-Sheng Chen, Leah E. Mechanic, Ben Racine, John Clarke, Lauren Clarke, Elizabeth Gillanders, Eric J. Feuer

**Affiliations:** ^1^Department of Genetics, The University of Texas MD Anderson Cancer Center, Houston, TX, 77030, ^2^Statistical Methodology and Applications Branch, Surveillance Research Program, Division of Cancer Control and Population Sciences, National Cancer Institute (NCI), National Institutes of Health (NIH), Bethesda, MD 20892, ^3^Host Susceptibility Factors Branch, Epidemiology and Genomics Research Program, Division of Cancer Control and Population Sciences, NCI, NIH, Bethesda, MD 20892 and ^4^Cornerstone Systems Northwest, Inc. Lynden, WA 98264

## Abstract

**Summary:** Many simulation methods and programs have been developed to simulate genetic data of the human genome. These data have been widely used, for example, to predict properties of populations retrospectively or prospectively according to mathematically intractable genetic models, and to assist the validation, statistical inference and power analysis of a variety of statistical models. However, owing to the differences in type of genetic data of interest, simulation methods, evolutionary features, input and output formats, terminologies and assumptions for different applications, choosing the right tool for a particular study can be a resource-intensive process that usually involves searching, downloading and testing many different simulation programs. Genetic Simulation Resources (GSR) is a website provided by the National Cancer Institute (NCI) that aims to help researchers compare and choose the appropriate simulation tools for their studies. This website allows authors of simulation software to register their applications and describe them with well-defined attributes, thus allowing site users to search and compare simulators according to specified features.

**Availability:** http://popmodels.cancercontrol.cancer.gov/gsr.

**Contact:** gsr@mail.nih.gov

## 1 INTRODUCTION

Owing to the cost and availability of genetic samples, lack of knowledge of causal variants that contribute to observed phenotypes and mathematical intractability of complex evolutionary models, computer simulations have been widely used, among many applications, to predict outcomes under realistic genetic scenarios (e.g. Peng and Kimmel, 2007), to compare and verify analytical methods or tools (e.g. Spencer *et al.*, 2009) and to estimate parameters of evolutionary models (e.g. Peter *et al.*, 2010). With increasing power of personal computers and the availability of computer clusters, novel simulation methods and sophisticated simulation programs have been and continue to be developed to simulate genetic data for new application areas such as large-scale genomic studies (Dalquen *et al.* 2012).

Despite the availability of a large number of simulation programs, choosing appropriate simulation programs for a particular research topic can be a time-consuming process that usually involves studying, downloading and testing many different tools with varying quality. Adding to the difficulties is the fact that many software applications lack comprehensive documentation, and use implicit assumptions and terminologies that are familiar only to researchers in particular research areas. As a result, at an NCI-sponsored conference, meeting participants recommended creating a web resource that summarizes available genetic simulation programs (Mechanic *et al.*, 2012).

Genetic Simulation Resources (GSR) is a website provided by NCI that aims to help researchers compare and choose the right simulation tools for their studies. This website allows authors of simulation software to register their applications and describe them with standardized attributes that are understandable to researchers in diverse research areas. Visitors of this website can browse a catalogue of genetic data simulators, review simulators of interest and search and compare simulators according to specified features. This pre-sorting allows researchers to focus on the most applicable simulators before starting the time-consuming process of downloading and testing the packages themselves.

## 2 METHODS

We searched published articles for software applications that simulate genetic data for the human genome in scientific journals such as *Bioinformatics*, *BMC Bioinformatics*, *Genetics* and *Molecular Biology and Evolution*. We selected simulators that can simulate genetic markers, haploid and diploid DNA sequences and RNA and protein sequences of the human genome. We excluded simulators without an accessible web page or download link and those that are designed for teaching purposes and are limited in their ability to simulate usable genetic data. We also excluded packages that have been replaced by newer or updated packages from the same authors.

We collected basic information of selected simulators, including short and long descriptions, URL to package web page, project start date and version and release date of the most recent release. We went through publications and documentation of these simulators and summarized their features with 167 attributes in 8 categories and 25 subcategories. These attributes range from key features such as type of genetic variations that can be simulated (e.g. single nucleotide polymorphism, insertion and deletion and microsatellite) and simulation methods (e.g. coalescent, forward time, resampling based and phylogenetic), to development features such as programming language, supported platform and license information. Because not all aspects of packages will be captured using these standard attributes, we allow package owners to annotate existing attributes with package-specific comments and define package-specific attributes.

We entered attributes of selected simulators and characterized them to the best of our knowledge. To ensure the accuracy of data, we sent a questionnaire to all package authors and received responses from approximately half of the authors, which may suggest that some packages have been left unmaintained for various reasons. We revised attributes of packages according to feedback from authors.

**Fig. 1.**
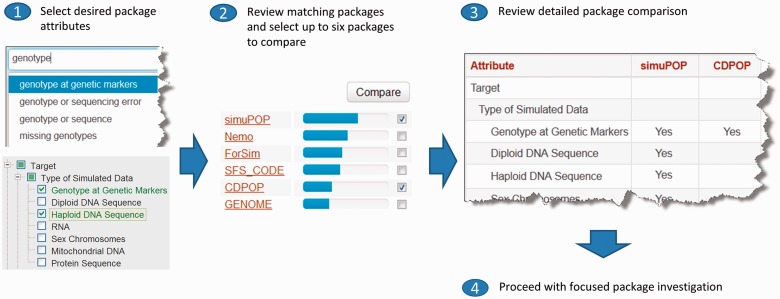
Illustration of the genetic simulation resources website

The GSR website currently provides an interface to a catalogue of 80 registered packages (Fig. 1), with a global search box, a list view of all software resources and interfaces to rank packages according to selected attributes and compare attributes of selected packages. Packages in this catalogue are continuously being added and updated by authors and users of simulation programs. GSR does not host or maintain individual packages and is not responsible for the accuracy and timely update of information related to these packages. We plan to evaluate the activity of packages regularly, based on factors including, but not limited to, availability of website and download links, number of updates and web visitors to package pages on GSR, number of applications (citations) and feedback from users of GSR. Packages that are no longer used by the research community will be phased out and eventually removed from GSR.

## 3 DISCUSSION

GSR provides a catalogue of genetic data simulators with detailed descriptions and list of features of each package, which make it easier for users of GSR to search and compare simulators and identify the most appropriate simulators for particular research topics. Package authors will also benefit from this service because a centralized catalogue would increase visibility of their software, and a clear list of features would help with documentation of their packages. GSR compliments existing review articles (e.g. Hoban *et al.*, 2012; Liu *et al.*, 2008) on genetic simulation programs by providing a comprehensive up-to-date list of programs, with links to web pages and searchable attributes, in a user-friendly format.

GSR is still under active development. Features that will be provided in the near future include an automated revision proposal and approval process, a citation management interface to track the applications of packages and a user-feedback system. We encourage all authors of genetic data simulators to register their packages in GSR and place a link to GSR on their websites, which would turn individually hosted packages to a web of simulators that could greatly facilitate the application, development and dissemination of genetic simulators.
